# Supplementary material for: Second harmonic generation imaging reveals entanglement of collagen fibers in the elephant trunk skin dermis
Source: Commun Biol. 2025 Jan 8;8:17. doi: 10.1038/s42003-024-07386-w (PMC11711191; doi:10.1038/s42003-024-07386-w)
Supplement: Supplementary file 1 — Supplementary Information [file 42003_2024_7386_MOESM1_ESM.pdf]

Supplemental Material

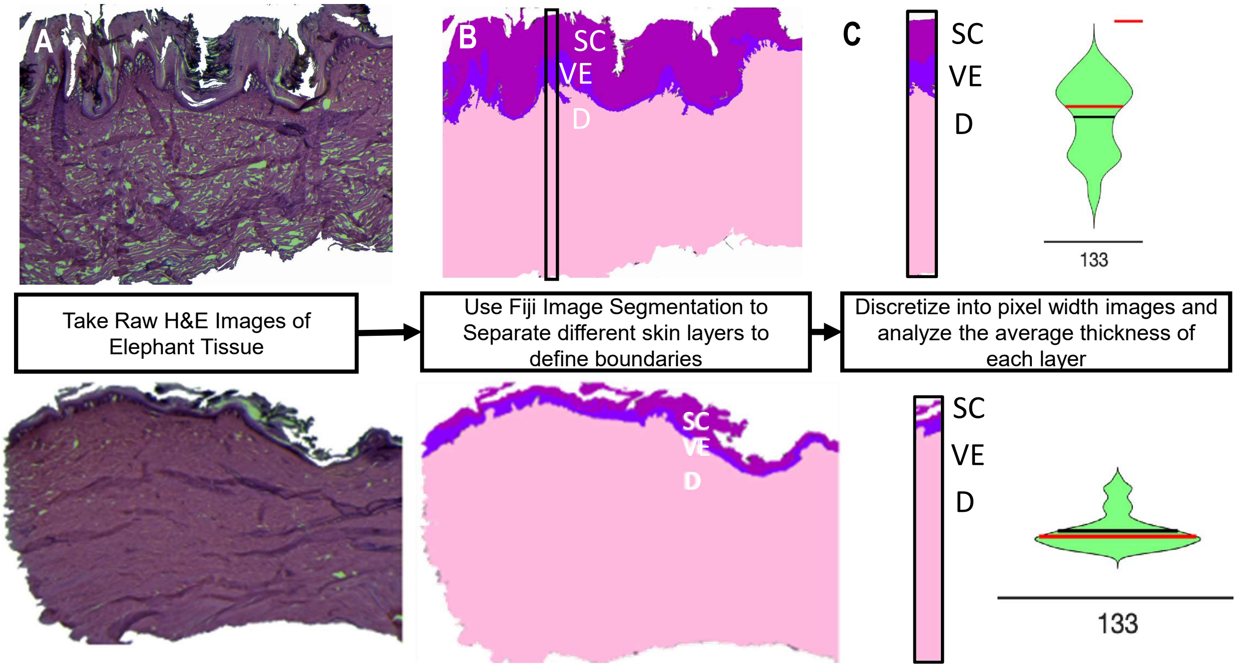

Supplementary Figure 1: Analysis of the staining technique performed on elephant tissue. A) Raw Hematoxylin & Eosin (H&E) stained tissue of the proximal dorsal (top) and proximal ventral (bottom). B) Images from part A that have been segmented using a Fiji segmentation macro, C) a one-pixel column length segment that is analyzed from B to obtain the violin plot. Red lines in the violin plot indicate media, with black lines indicating mode.

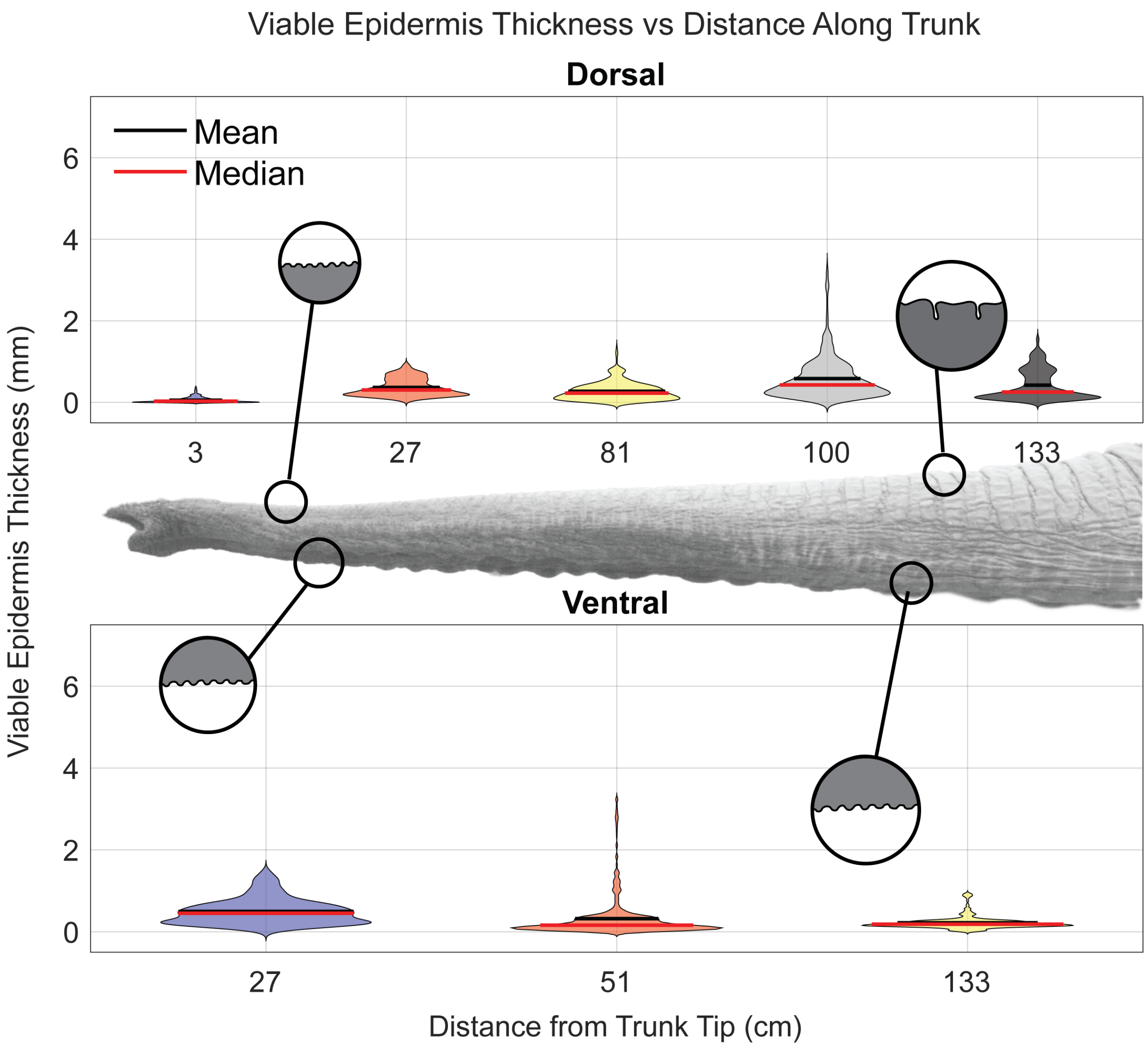

Supplementary Figure 2: Relationship between viable epidermis (VE) thickness and position on the trunk. The position is the distance from the trunk tip in cm.

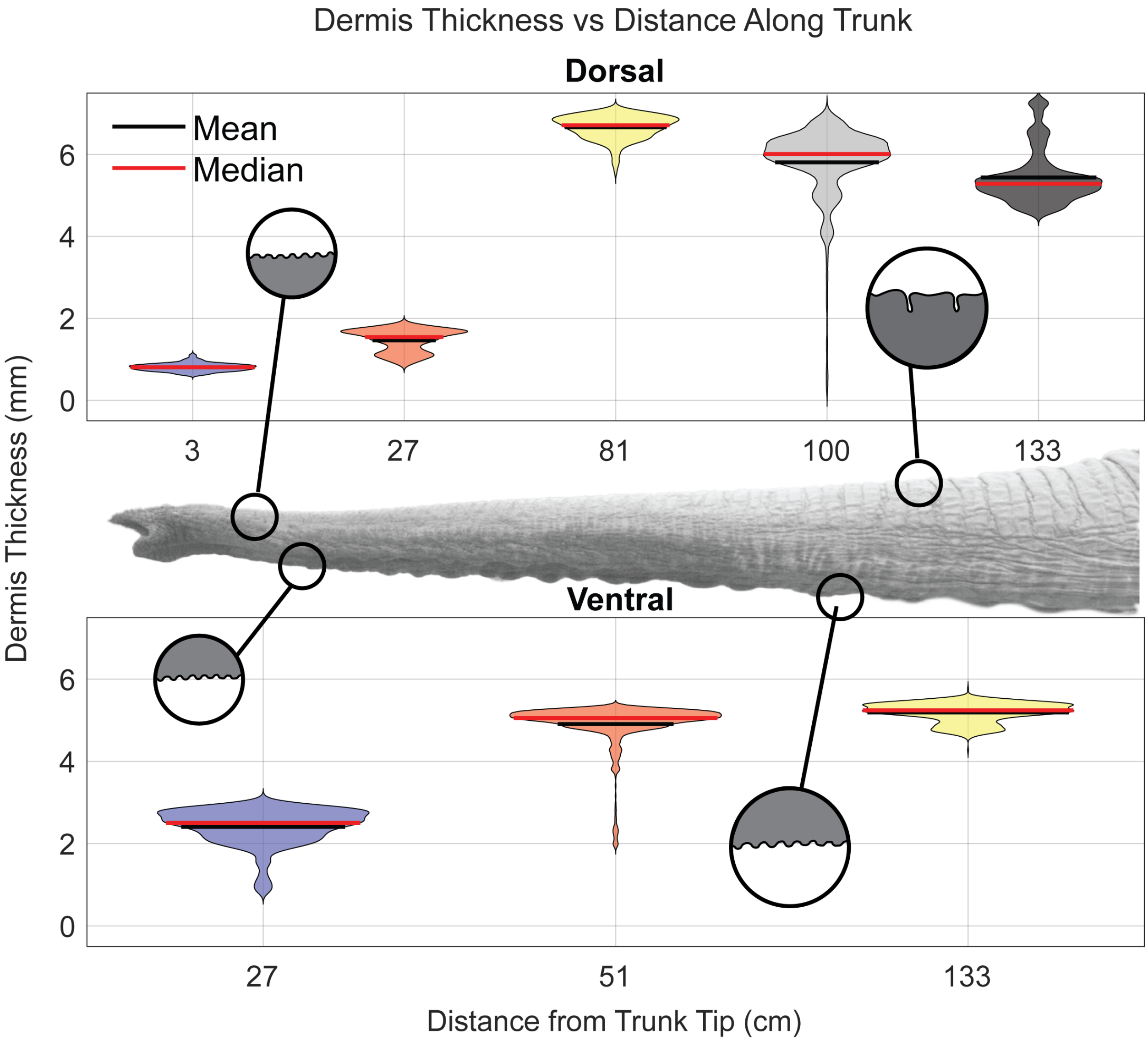

Supplementary Figure 3: Relationship between dermis (D) thickness and position on the trunk. The position is the distance from the trunk tip in cm.

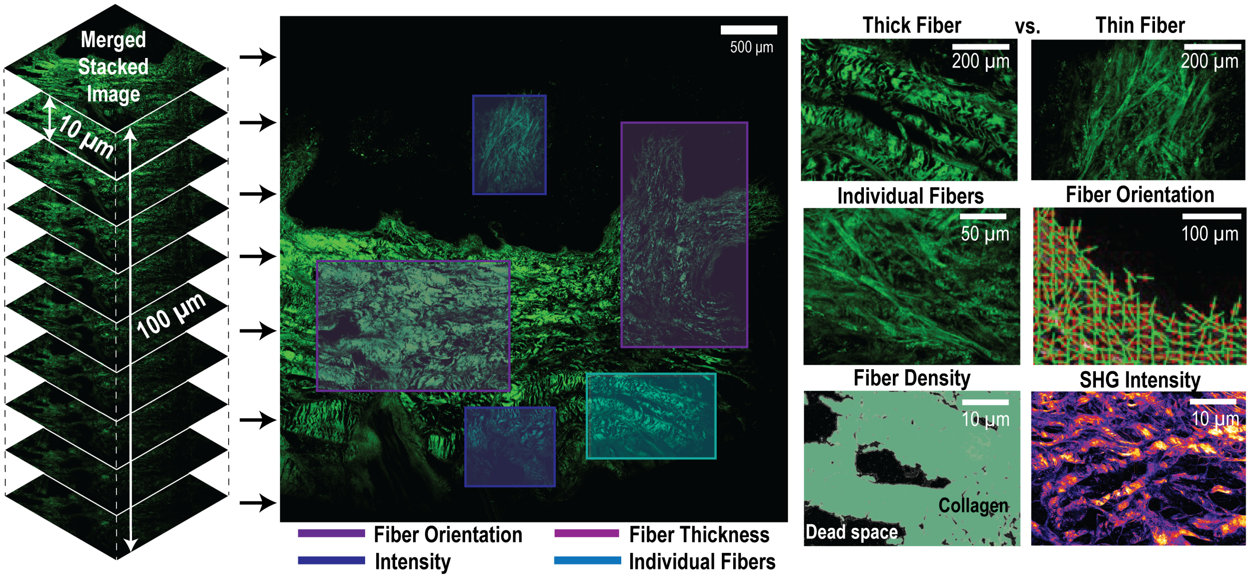

Supplementary Figure 4: Second Harmonic Generation (SHG) microscopy technique utilized for analysis of compositional differences in the elephant skin. Raw image stack separated by 10 μm stacked together to generate the compiled image. On the compiled imaged sections are highlighted to show the different analyses that can be performed from the stacked image generated in part A.
